# Effect of oral alpha-lipoic acid (ALA) on sperm parameters: a systematic review and meta-analysis

**DOI:** 10.1186/s12610-022-00173-9

**Published:** 2022-12-08

**Authors:** Liang Dong, Fang Yang, Junjun Li, Yulin Li, Xujun Yu, Xiaojin Zhang

**Affiliations:** 1 TCM Regulating Metabolic Diseases Key Laboratory of Sichuan Province, Hospital of Chengdu University of Traditional Chinese Medicine, Chengdu, People’s Republic of China; 2 Department of Andrology, The Reproductive & Women-Children Hospital, Chengdu University of Traditional Chinese Medicine, Chengdu, People’s Republic of China; 3 Chengdu University of Traditional Chinese Medicine, No.1166, Liutai Avenue, Wenjiang District, Chengdu, 611137 Sichuan People’s Republic of China

**Keywords:** Alpha-lipoic acid (ALA), Male infertility, Sperm, Meta-analysis, Systematic review, Acide alpha-lipoïque (ALA), Infertilité masculine, Sperme, Méta-analyse, Revue systématique

## Abstract

**Background:**

Male fertility has gradually become a worldwide problem with limitations in the treatment. Alpha-lipoic acid, has been applied to improve the quality of sperm in clinical practice. However, there was currently no high quality of systematic review to evaluate the effects of alpha-lipoic acid on sperm parameters.

**Material and methods:**

The Cochrane Library, MEDLINE, EMBASE, Web of Science, Clinicaltrials.org, China National Knowledge Infrastructure Database, China Biology Medicine Database, etc., were retrieved. Related randomized controlled trials had be collected and selected up to March 10, 2022. English literature and Chinese literature were searched using terms including “male infertility”, “semen”, “sperm”, “alpha-lipoid acid”, “α-lipoid acid”, “alpha lipoid acid”, “thioctic acid”. All statistical analyses were conducted by RevMan 5.3.

**Results:**

A total of 133 participants in three studies included. Compared with sham therapy, treated with alpha-lipoic acid has significant improvement in the following sperm parameters, including abnormal sperm forms (mean difference[MD] = -1.06, 95% confidence interval [CI] = -1.29–0.84, *p* < 0.00001), sperm concentration (MD = 3.98, 95%CI = 2.28–5.67, *p* < 0.00001), sperm total motility (grade a+b+c) (MD = 6.68, 95%CI = 4.88–8.48, *p* < 0.00001) and progressive motility(grade a+b) (MD = 6.90, 95%CI = 5.62–8.17, *p* < 0.00001) and semen volume(MD = -0.17, 95%CI = -0.31–0.02, *p* = 0.03).

**Conclusions:**

In this meta-analysis of three randomized controlled trials, compared with other treatments, alpha-lipoic acid could improve normal sperm forms, sperm concentration, sperm total motility and progressive motility, but more stringent randomized controlled trials must be conducted.

## Résumé

**Contexte:**

La fertilité masculine est progressivement devenue un problème mondial avec des possibilités de traitement limitées. L’acide alpha-lipoïque a été utilisé dans la pratique clinique pour améliorer la qualité du sperme. Cependant, il n’existe actuellement aucune revue systématique de haute qualité sur l’évaluation des effets de l’acide alpha-lipoïque sur les paramètres du sperme.

Les bases de données Cochrane, MEDLINE, EMBASE, Web of Science, Clinicaltrials.org, China National Knowledge Infrastructure Database, China Biology Medicine Database, etc., ont été utilisées. Les essais contrôlés randomisés en relation ont été recueillis et sélectionnés jusqu’au 10 mars 2022.  La littérature anglaise et la littérature chinoise ont été recherchées en utilisant les termes tels que « infertilité masculine », « sperme », « spermatozoïde », « acide alpha-lipoïque », « acide α-lipoïque », « acide alpha lipoïque », « acide  thioctique ». Toutes les analyses statistiques ont été effectuées par RevMan 5.3.

**Résultats:**

Au total, 133 participants provenant de trois études ont été inclus. Par rapport à la thérapie simulée, le traitement par l’acide alpha-lipoïque présente une amélioration significative des paramètres de sperme qui suivent : formes anormales de spermatozoïdes (différence moyenne [DM] = -1,06 ; intervalle de confiance à 95 % [IC] = -1,29--0,84, p < 0,00001), concentration de spermatozoïdes (DM = 3,98 ; IC à 95 % = 2,28-5,67, p < 0,00001), motilité totale des spermatozoïdes (grade a + b + c) (MD = 6,68 ; IC à 95% = 4,88-8,48, p < 0,00001), motilité progressive (grade a + b) (MD = 6,90 ; IC à 95% = 5,62-8,17, p <0.00001) et volume de sperme (MD =-0,17 ; IC à 95 % = -0,31--0,02, p = 0,03).

**Conclusions:**

Dans cette méta-analyse de trois essais contrôlés randomisés, l’acide alpha-lipoïque, par rapport à d’autres traitements, pourrait améliorer les formes normales de spermatozoïdes, la concentration de spermatozoïdes, la motilité totale des spermatozoïdes et la motilité progressive ;  mais des essais contrôlés randomisés plus rigoureux doivent être menés.

## Introduction

More than 15% married couples in the world suffer from fertility problems, of which about 50% are caused by men [1]. In China, the quality of male sperm decreases by 1% every year [2]. The drugs for the treatment of male infertility are very limited. At present, one of the commonly used drugs are antioxidants [3], including acetyl-L-carnitine, L-carnitine fumarate, N-acetylcysteine, Glutathione, Vitamins E, Vitamins C, Carnitines, Coenzyme-Q10, Selenium, Zinc, Folic Acid, etc. Among them, alpha-lipoic acid (ALA), considered as a powerful biological antioxidant and used for preventing metabolic and reproductive changes in diabetes patients [4], is also currently the most effective lipid- and water-soluble antioxidant. It can maintain sperm motility and vitality by reducing the production of reactive oxygen species (ROS), and also protect sperm DNA integrity [5]. Many clinical and animal studies had shown that ALA can improve sperm parameters and reduce sperm DNA damage, so as to improve male fertility [6, 7], also during sperm preparation process [5].

So far, there was only one systematic review about ALA in the treatment of male infertility, which included various types of researches [8], and not included in the latest research results. Based on the protocol we did in the early stage [9], we continue to do this work. At all, we sought to provide high level evidence-based medical evidence for urologists and andrologists to make clinical decisions for male infertility treatment. This study was registered on PROSPERO. Registration number: PROSPERO CRD42019145592.

## Materials and methods

### Search strategy

**Fig. 1:**
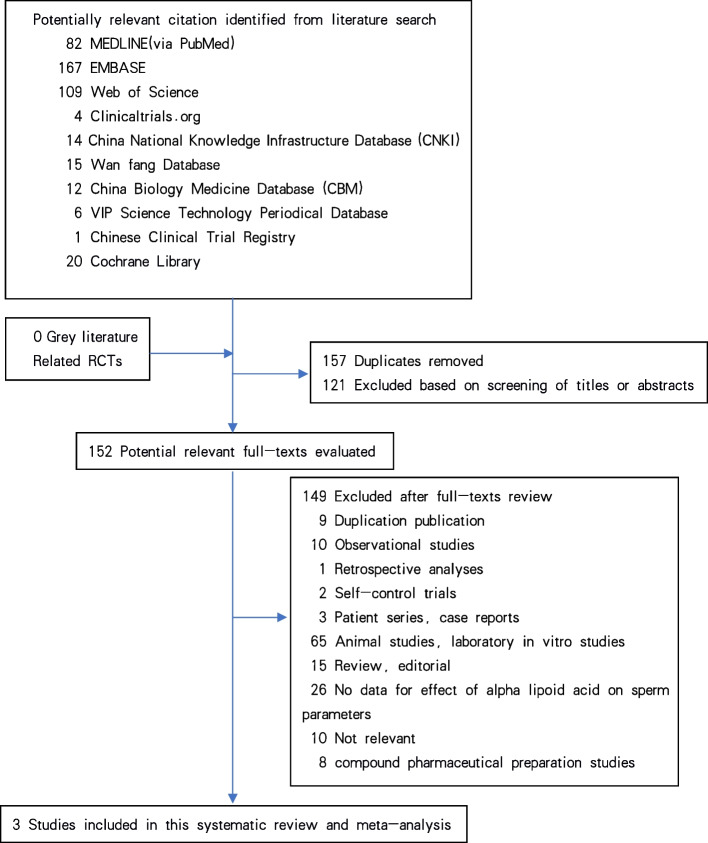
Flow diagram for study selection

The electronic databases of Cochrane Library, MEDLINE(via PubMed), Web of Science, EMBASE, Clinicaltrials.org., China Biology Medicine Database (CBM), China National Knowledge Infrastructure Database (CNKI), Wan fang Database, VIP Science Technology Periodical Database and Chinese Clinical Trial Registry were retrieved. Grey literature had be searched in Open Grey. Related randomized controlled trials (RCTs) had be searched and selected up to March 10, 2022. As of this month’s submission, we have retrieved and updated the data again. According to the inclusion criteria, the search subject terms were determined with the PICO principle, and then the corresponding electronic search was carried out. We chose medical subject heading and text key words “male infertility” or “sperm” or “semen” AND “oral alpha-lipoic acid” or “alpha-lipoid acid” or “alpha lipoic acid” or “α-lipoid acid” or “thioctic acid”, and different search strategies to fit different databases. Chinese form of the above terms will be used in Chinese search. This systematic review and meta-analysis have carried out in strict accordance with the guidelines of Preferred Reporting Items for Systematic Reviews and Meta-Analyses (PRISMA) [10]. A flow diagram for study selection is presented in Fig. 1.

### Inclusion criteria and trial selection

Only RCTs about male infertility treating with ALA were be included in the study, and sperm quality analysis was performed following the World Health Organization (WHO) guidelines [11, 12]. In similar patients and studies using the same method, only the one with largest sample size or recently studies was included. The following studies were excluded: non-human studies, editorial, conference proceedings, literature reviews. When two or more studies were conducted based on the same RCT participants, only the study with the most recently updated data was included. Any disagreements were resolved by consensus.

### Data extraction

Related data in the included articles was extracted independently by three investigators (Liang Dong, Fang Yang and Junjun Li) according to the PRISMA statement, and all discrepancies were resolved through adjudication and discussion by the other reviewer (Yulin Li). The words in abstracts such as "randomized" or "quasi-randomized" were used in all studies, regardless of whether they were blind or not. For each study, the following information were extracted: author, publication time, country, sample size, treatment course, edition of WHO guidelines, inclusion criteria, outcome indicators, treatment group medicine & dosage and post-treatment data. Study investigators from Raaia et al.’s study [13] were contacted to obtain further information, but the researchers did not reply to our e-mail.

### Quality assessment

The items of randomization, method to generate the sequence of randomization, randomization concealment, blinding, results data integrity, selective outcome reports and other potential bias sources in the included RCTs were assessed by the Cochrane Risk of Bias Assessment tool [14]. Graph and summary about risk of bias were produced with RevMan 5.3 [15]. All the domains were independently assessed by two trained investigators (Liang Dong, Xiaojin Zhang). All the disputes were resolved by a third professional reviewer through discussion and adjudication.

### Data synthesis and analysis

The mean values of sperm parameters after treatment with ALA and sham-therapy in each study were collected, including semen volume, sperm concentration, total sperm number, sperm normal forms, abnormal sperm forms, fast progressively motility(grade a), slow progressively motility(grade b), non-progressively motility(grade c), immotile spermatozoa(grade d), total motility(grade a+b+c), progressive motility(grade a+b) and vitality. Statistics analyses were estimated with RevMan 5.3 and displayed as a forest plot, while a funnel plot has been generated to assess the risk of bias. Statistical tests were two-sided and used *p*-value less than 0.05 as a significance threshold. The Egger's test (by Stata software) was used for investigating publication bias to small study effects when necessary [16, 17]. The heterogeneity between studies was assessed by standard X^2^ test and I^2^ statistics [18]. The purpose of subgroup analysis was to study interaction or effect modification, which was used to answer questions about specific patients, intervention types or study types. Sensitivity analysis was conducted by excluding the effects of individual studies one by one on the overall estimates, that was used to evaluate whether the results of meta-analysis were stable and reliable [19].

## Results

### Study characteristics

**Fig. 2:**
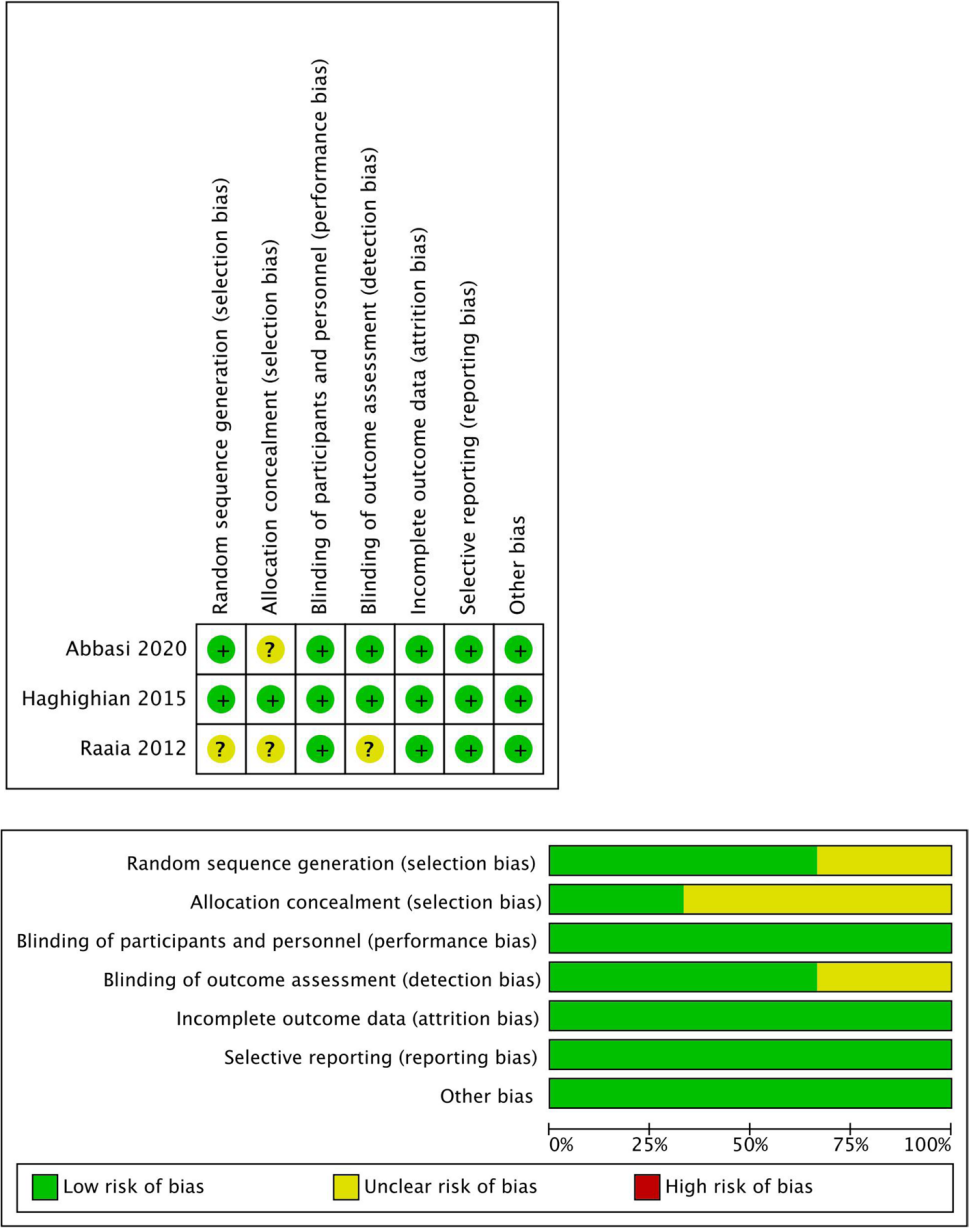
The risk of bias of included studies

Three RCTs involving 133 participants completed were included in this meta-analysis (Tables 1 and 2). One study [13] was conducted in Egypt, anther two [20, 21] took place in Iran. All three studies used sham therapy in the control group by using probes that looked and tasted similar to the active treatment probes. Oral ALA 600 mg per day was used in all three studies. One study [13] was 300 mg twice a day, and the other two studies [20, 21] were 600mg once a day. The treatment courses of the three studies were 3 months [13], 12 weeks [21] and 80 days [20] respectively. Although there was a difference of treatment course, the difference was very small, which was roughly the same as one spermatogenic cycle, so it did not affect our statistical results. Two studies [13, 20] used the 2010 version of WHO semen examination results guidelines, only one study [21] used the 1992 and 1999 WHO guidelines. The semen parameters counted in these three studies including sperm concentration, semen volume, total sperm number, normal forms, abnormal sperm forms, fast progressively motility(grade a), slow progressively motility(grade b), non-progressively motility(grade c), immotile spermatozoa(grade d), total motility(grade a+b+c), progressive motility(grade a+b), vitality, sperm lipid per-oxidation, sperm lipid per-oxidation (intensity), DNA fragmentation, DNA damage index, sperm protamine deficiency and pregnancy rate, which were listed in the Tables 1 and 2. As for the inclusion criteria of the three studies, in one study [20], patients with varicocele were randomly divided into two groups after operation with ALA and sham treatment. But interestingly, most of the sperm parameters in the preoperative and postoperative had no significant difference between the ALA group and the control group. Further information of inclusion diagnostic criteria were listed in Tables 1 and 2. For all of the included studies, the risk of bias were low. However, the risk of bias was unclear for several domains, Fig. 2 showed that 33.3% of the studies had an unclear risk of bias in randomization, and only 66.6% of studies had good blinding.

**Table 1:** Characteristics of the included studies of ALA on sperm parameters in this systematic review

**Basic Information**	**Study Design**	**Information of the Treatment/Control groups**				**Diagnostic Criteria**	**Outcome Indicatorss and Edition of WHO guidelines**	
		**Treatment groups** **Number of inclusion (completed)**	**Control groups** **Number of inclusion (completed)**	**Treatment** **Course**	**Treatment groups Medicine & dosage**		**Outcome Indicators(unites)**	**Edition of WHO guidelines(year)**
Raaia et al. [13] 2012 Egypt	Placebo-controlled Double-blind	30(24)	30(24)	3 months	oral ALA tablets at a dose of 300 mg twice/day (Thiotacid 300 mg; Eva Company, Cairo, Egypt)	History of infertility for more than 1 year, sperm concentration more than 5 million sperms/ ml, low motility: less than 32% progressive motility and less than 40% progressive and nonprogressive motility, no clinical or duplex evidence of varicocele, no evidence of genitourinary tract infection, no clinical symptoms of endocrinal or genetic disorders (e.g. Klinefelter’s syndrome), normal serum follicle-stimulating hormone and testosterone levels, no history of medical diseases (e.g. diabetes, hypertension, liver, or kidney diseases), no concurrent intake of fertility-enhancing medication, and no history of chemotherapeutic medications.	sperm concentration(10^6^/ml) total motility(grade a+b+c) (%) progressive motility(grade a+b) (%) abnormal sperm forms (%) pregnancy rate (%)	fifth edition(2010)
Haghighian et al. [21] 2015 Iran	Randomized Triple-blind Placebo-controlled	24(23)	24(21)	12 weeks	600 mg ALA once daily	unwilling childlessness at least 24 months in duration with a female partner, no medical condition that could account for infertility, and a normal fertile female partner according to investigations. All patients were needed to have stopped all medical therapy ≥ 12 weeks before study initiation Exclusion criteria included the history of epididymo-orchitis, prostatitis, genital trauma, testicular torsion, inguinal or genital surgery, urinary tract infection, or previous hormonal therapy; another genital disease (cryptorchidism, current genital inflflammation or varicocele); severe general or central nervous system disease and endocrinopathy; use of cytotoxic drugs, immunosuppressants, anticonvulsants, androgens, or antiandrogens; and a recent history of sexually transmitted infection. Patients were also excluded from analysis if they had psychologic or physiologic abnormalities that would impair sexual performance or the ability to provide semen samples; drug or alcohol abuse; hepatobiliary disease; significant renal insuffificiency; occupational and environmental subjections to possible reproductive toxins; a body mass index of ≥ 30 kg/m2; participation in another investigational study; and unlikely availability for follow-up	semen volume(ml) total sperm number(10^6^/ejaculate) sperm concentration(10^6^/ml) progressive motility(grade a+b) (%) fast progressively motility(grade a) (%) slow progressively motility(grade b) (%) non-progressively motility(grade c) (%) immotile spermatozoa(grade d) (%) total motility(grade a+b+c) (%) normal forms(%) vitality(%)	forth editon(1999)
Abbasi et al. [20] 2020 Iran	Triple-blind Randomized Placebo-controlled	30(19)	30(22)	80 days	daily doses of 600 mg of ALA (Raha, Iran)	A total of 60 men aged 19 to 45 years, with uni/bilateral grade II–III varicocele (confirmed by Doppler duplex ultrasonography if ambiguous on palpation) met the inclusion criteria and were enrolled in the study Individuals with azoospermia, occupational exposure to heat, radiation, and pesticides, a history of mumps, cryptorchidism, solitary testis, urogenital malignancies/infections, endocrinopathies, Sertoli cellonly syndrome, leukocytospermia, scrotal trauma, high fever prior to sampling, recurrent varicocele, severe alcoholism and heavy smoking were not included in this study	sperm concentration (10^6^/ml) semen volume (ml) abnormal sperm forms (%) total motility(grade a+b+c) (%) progressive motility(grade a+b) (%) sperm lipid peroxidation(%) spermlipid peroxidation(intensity) DNA fragmentation(%) DNA damage index(%) sperm protamine deficiency(%)	fifth edition(2010)

*Legend:* Three RCTs with 133 participants completed were included. One study was conducted in Egypt, anther two took place in Iran. All three studies used sham therapy in the control group, while oral ALA 600 mg per day was used in treatment group. The treatment courses of the three studies were 3 months, 12 weeks and 80 days respectively. Two studies used the 2010 version of WHO semen examination results. The inclusion and exclusion criteria of the three studies were also clearly shown in the table

*ALA* Alpha-lipoic acid

**Table 2:** The results of ALA and sham therapy in two groups of the three studies

Basic Information	Results(Mean ± SD) after treatment in two groups		
	**treatment group(with ALA)**	**control group(with sham therapy)**	***P* Value**
Raaia et al. [13] 2012 Egypt	**1.Semen parameters after one month** sperm concentration(10^6^/ml): 40.4 ± 86.7 total motility(grade a+b+c) (%): 39.2 ± 20.3 progressive motility(grade a+b) (%): 27.6 ± 16.6 **2.Semen parameters after two months** sperm concentration(10^6^/ml): 43.7 ± 39.7 total motility(grade a+b+c) (%): 45.1 ± 17.9 progressive motility(grade a+b) (%): 33.3 ± 15.8 **3.Semen parameters after three months** sperm concentration(10^6^/ml): 40.0 ± 27.6 total motility(grade a+b+c) (%): 46.9 ± 19.8 progressive motility(grade a+b) (%): 34.0 ± 17.8 abnormal sperm forms (%): 63.7 ± 13.3	**1.Semen parameters after one month** sperm concentration(10^6^/ml): 26.6 ± 27.4 total motility(grade a+b+c) (%): 40.6 ± 20.1 progressive motility(grade a+b) (%): 29.9 ± 19.9 **2.Semen parameters after two months** sperm concentration(10^6^/ml): 26.7 ± 22.5 total motility(grade a+b+c) (%): 39.7 ± 16.2 progressive motility(grade a+b) (%): 28.2 ± 14.5 **3.Semen parameters after three months** sperm concentration(10^6^/ml): 21.8 ± 17.9 total motility(grade a+b+c) (%): 42.8 ± 19.9 progressive motility(grade a+b) (%): 29.8 ± 17.9 abnormal sperm forms (%): 69.3 ± 17.5	**1.Semen parameters after one month** sperm concentration: 0.216 total motility(grade a+b+c): 0.680 progressive motility(grade a+b): 0.789 **2.Semen parameters after two months** sperm concentration: 0.042* total motility(grade a+b+c): 0.490 progressive motility(grade a+b): 0.415 **3.Semen parameters after three months** sperm concentration: 0.001* total motility(grade a+b+c): 0.288 progressive motility(grade a+b): 0.303 abnormal sperm forms: 0.128
Haghighian et al. [21] 2015 Iran	semen volume(ml): 3.5 ± 0.3 total sperm number(10^6^/ejaculate): 90.4 ± 6.2 sperm concentration(10^6^/ml): 26.3 ± 3.1 progressive motility(grade a+b) (%): 33.4 ± 2.9 fast progressively motility(grade a) (%): 6.5 ± 2.2 slow progressively motility(grade b) (%): 26.9 ± 2.4 non-progressively motility(grade c) (%): 7.1 ± 3.8 immotile spermatozoa(grade d) (%): 59.3 ± 4.5 total motility(grade a+b+c) (%): 40.6 ± 4.9 normal forms(%): 15.3 ± 3.6 vitality(%): 71.4 ± 3.5	semen volume(ml): 3.5 ± 0.3 total sperm number(10^6^/ejaculate): 77.5 ± 4.5 sperm concentration(10^6^/ml): 22.8 ± 2.7 progressive motility(grade a+b) (%): 27.1 ± 2.3 fast progressively motility(grade a) (%): 2.7 ± 1.3 slow progressively motility(grade b) (%): 24.3 ± 2.2 non-progressively motility(grade c) (%): 8.8 ± 3.2 immotile spermatozoa(grade d) (%): 63.9 ± 2.9 total motility(grade a+b+c) (%): 36.0 ± 3.1 normal forms(%): 13.8 ± 3.7 vitality(%): 72.8 ± 4	semen volume: 0.991 total sperm number: < 0.001* sperm concentration: < 0.001* progressive motility(grade a+b): < 0.001* fast progressively motility(grade a): < 0.001* slow progressively motility(grade b): 0.011* non-progressively motility(grade c): 0.122 immotile spermatozoa(grade d): 0.005* total motility(grade a+b+c): 0.004* normal forms: 0.153 vitality: 0.255
Abbasi et al. [20] 2020 Iran	**1.before microsurgical repair of varicocele/no-medication** sperm concentration (10^6^/ml): 52.3 ± 12.5 semen volume (ml): 1.9 ± 0.2 abnormal sperm forms (%): 97.2 ± 0.3 total motility(grade a+b+c) (%): 36.4 ± 5.6 progressive motility(grade a+b) (%): 23.7 ± 3.4 sperm lipid peroxidation(%): 40.7 ± 3.3 spermlipid peroxidation(intensity): 25.2 ± 3.0 DNA fragmentation(%): 11.2 ± 0.6 DNA damage index(%): 23.2 ± 1.5 sperm protamine deficiency(%): 35.3 ± 3.2 **2. post-varicocelectomy/post-medication semen parameters** sperm concentration (10^6^/ml): 81.6 ± 16.1 semen volume (ml): 3.1 ± 0.4 abnormal sperm forms (%): 93.4 ± 0.7 total motility(grade a+b+c) (%): 50.3 ± 5.2 progressive motility(grade a+b) (%): 35.7 ± 3.9 sperm lipid peroxidation(%): 22.6 ± 1.6 spermlipid peroxidation(intensity): 21.7 ± 1.8 DNA fragmentation(%): 12.2 ± 1.0 DNA damage index(%): 18.3 ± 1.4 sperm protamine deficiency(%): 33.5 ± 3.2	**1.before microsurgical repair of varicocele/no-medication** sperm concentration (10^6^/ml): 47.9 ± 12.1 semen volume (ml): 2.3 ± 0.3 abnormal sperm forms (%): 98.2 ± 0.3 total motility(grade a+b+c) (%): 38.3 ± 5.7 progressive motility(grade a+b) (%): 24.8 ± 4.0 sperm lipid peroxidation(%): 36.2 ± 3.3 spermlipid peroxidation(intensity): 24.1 ± 1.7 DNA fragmentation(%): 13.6 ± 2.4 DNA damage index(%): 20.4 ± 2.3 sperm protamine deficiency(%): 41.7 ± 4.1 **2. post-varicocelectomy/post-medication semen parameters** sperm concentration (10^6^/ml): 74.4 ± 12.7 semen volume (ml): 3.6 ± 0.3 abnormal sperm forms (%): 95.4 ± 0.7 total motility(grade a+b+c) (%): 39.7 ± 4.4 progressive motility(grade a+b) (%): 26.7 ± 3.8 sperm lipid peroxidation(%): 24.0 ± 1.8 spermlipid peroxidation(intensity): 21.3 ± 1.5 DNA fragmentation(%): 10.3 ± 0.9 DNA damage index(%): 16.4 ± 1.2 sperm protamine deficiency(%): 37.4 ± 3.6	**1.before microsurgical repair of varicocele** sperm concentration: 0.8 semen volume: 0.45 abnormal sperm forms: 0.04* total motility(grade a+b+c): 0.8 progressive motility(grade a+b): 0.83 sperm lipid peroxidation: 0.34 spermlipid peroxidation(intensity): 0.72 DNA fragmentation: 0.39 DNA damage index: 0.32 sperm protamine deficiency: 0.24 **2. post-varicocelectomy/post-medication semen parameters** sperm concentration: 0.72 semen volume: 0.42 abnormal sperm forms: 0.056 total motility(grade a+b+c): 0.12 progressive motility(grade a+b): 0.11 sperm lipid peroxidation: 0.56 spermlipid peroxidation(intensity): 0.84 DNA fragmentation: 0.2 DNA damage index: 0.32 sperm protamine deficiency: 0.42

*Legend:* *This parameter had significant changes after ALA treatment, compared with the control group. Statistical tests were two-sided with T test and used *p*-value less than 0.05 as a significance threshold

*ALA* Apha-lipoic acid

### Effect of ALA on abnormal sperm forms under WHO guidelines

**Fig. 3:**
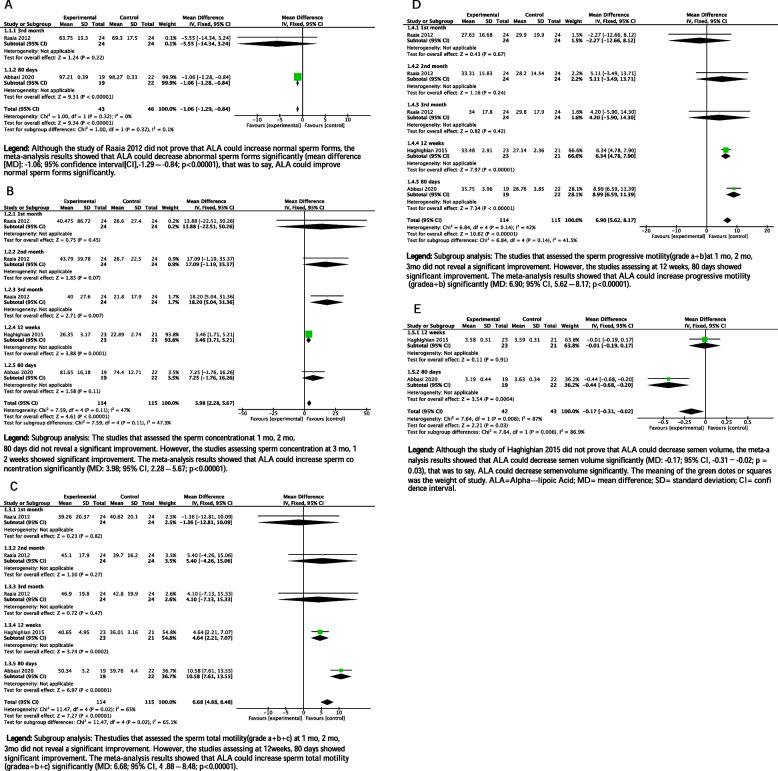
Clinical outcomes

Only two studies listed data on abnormal sperm forms, and there was a statistically significant decreased treated with ALA compared with those receiving sham therapy in abnormal sperm forms (MD: -1.06 points; 95% CI [-1.29, -0.84]; *p* < 0.00001; I^2^ = 0%, See in Fig. 3A).

### Effect of ALA on sperm concentration under WHO guidelines

Statistical analysis of oral ALA in different treatment courses showed that the sperm concentration was also statistically significant improvement compared with those receiving sham therapy (MD: 3.98 points; 95% CI [2.28, 5.67]; *p* < 0.00001; I^2^ = 47%, See in Fig. 3B).

The sensitivity analysis (see in Table 3) showed that, Raaia 2012 [13] (3rd month) and Haghighian 2015 [19] were found to affect the overall prevalence estimate by an absolute difference for the indicator.

**Table 3:** Sensitivity analysis of sperm concentration

Study	Mean Difference	Lower CI	Upper CI	*P*	I^2^
Omitting Raaia 2012 1st month	3.95	[2.26;	5.65]	< 0.00001	59%
Omitting Raaia 2012 2nd month	3.86	[2.16;	5.56]	< 0.00001	46%
Omitting Raaia 2012 3rd month	3.74	[2.03;	5.44]	< 0.0001	1%
Omitting Haghighian 2015	11.72	[4.95;	18.49]	0.0007	0%
Omitting Abbasi 2020	3.86	[2.13;	5.58]	< 0.0001	58%
**Pooled estimate**	**3.98**	**[2.28;**	**5.67]**	** < 0.00001**	**47%**

*Legend:* Sensitivity analysis showed that Raaia 2012 3rd month and Haghighian 2015 were found to affect the overall prevalence estimate by an absolute difference for the indicator

*CI* Confidence interval

### Effect of ALA on sperm total motility(grade a+b+c) under WHO guidelines

There was a statistically significant improved treated with ALA compared with those receiving sham therapy in sperm total motility(a+b+c) (MD: 6.68 points; 95% CI [4.88, 8.48]; *p* < 0.00001; I^2^ = 65%, See in Fig. 3C).

The sensitivity analysis (see in Table 4) showed that, Abbasi 2020 [20] was found to affect the overall prevalence estimate by an absolute difference for the indicator.

**Table 4:** Sensitivity analysis of sperm total motility(grade a+b+c)

Study	Mean Difference	Lower CI	Upper CI	*P*	I^2^
Omitting Raaia 2012 1st month	6.89	[5.06;	8.71]	< 0.00001	69%
Omitting Raaia 2012 2nd month	6.73	[4.89;	8.56]	< 0.00001	74%
Omitting Raaia 2012 3rd month	6.75	[4.93;	8.58]	< 0.00001	73%
Omitting Haghighian 2015	9.16	[6.48;	11.84]	< 0.00001	45%
Omitting Abbasi 2020	4.43	[2.16;	6.69]	0.0001	0%
**Pooled estimate**	**6.68**	**[4.88;**	**8.48]**	** < 0.00001**	**65%**

*Legend:* Sensitivity analysis showed that Abbasi 2020 was found to affect the overall prevalence estimate by an absolute difference for the indicator

*CI* Confidence interval

### Effect of ALA on progressive motility(grade a+b) under WHO guidelines

There was a statistically significant improvement treated with ALA compared with those receiving sham therapy in sperm progressive motility(grade a+b) (MD: 6.90 points; 95% CI [5.62, 8.17]; *p* < 0.00001; I^2^ = 42%, See in Fig. 3D).

### Effect of ALA on semen volume under WHO guidelines

Statistical analysis of the data of the two studies found that the amount of semen volume decreased after ALA treatment compared with those receiving sham therapy, which was statistically significant (MD: -0.17 points; 95% CI [-0.31, -0.02]; *p* = 0.03; I^2^ = 87%, See in Fig. 3E).

Because only two studies have made statistics of this data, sensitivity analysis did not necessary to be carried out.

### Assessment of publication bias

**Fig. 4:**
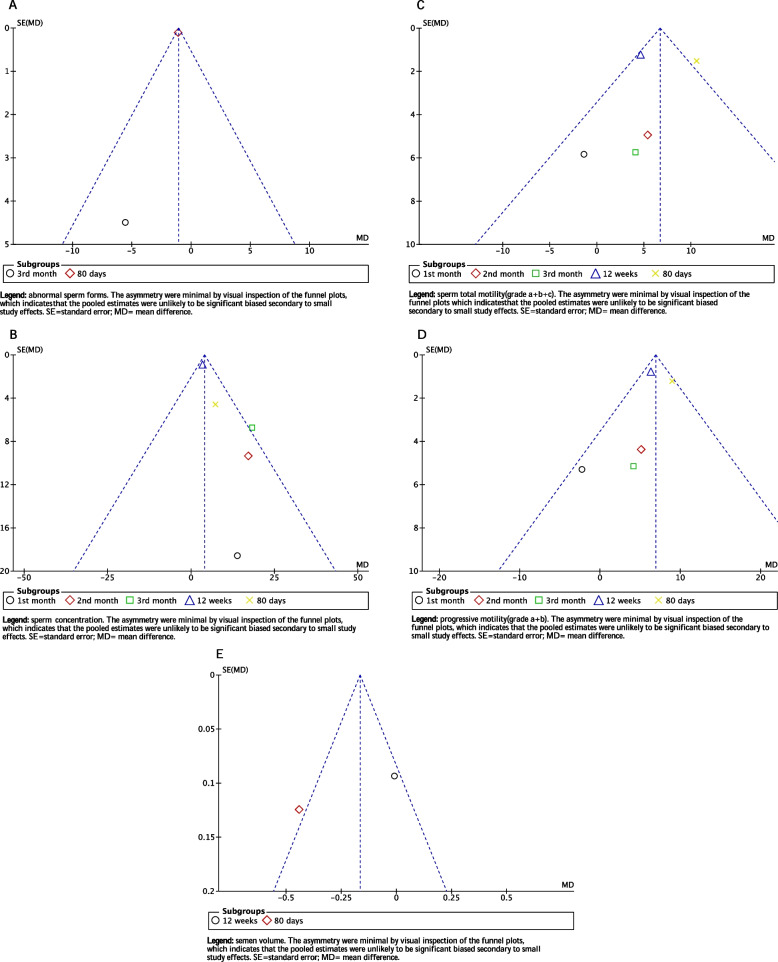
Funnel plots of this meta-analysis on sperm parameters

Although only three studies were included in the meta-analysis, funnel plots were drawn. The asymmetry were minimal by visual inspection of the funnel plots in the abnormal sperm forms, sperm concentration, sperm total motility(grade a+b+c), sperm progressive motility(grade a+b) and semen volume, which indicates that the pooled estimates were unlikely to be significant biased secondary to small study effects (Fig. 4).

## Discussion

Due to the great variability of infertility reason, the true incidence of male infertility is unknown, and the treatment options are also diverse [3]. Antioxidants are the most commonly used drugs in the treatment of male infertility. ALA, as one of the most powerful biological antioxidants, has a certain effect in improving the quality of male sperm [5].

Three RCTs involving 133 men in this systematic review and meta-analysis showed that with ALA, abnormal sperm forms, sperm concentration, sperm total motility, progressive motility(grade a+b) were significantly improved. However, the outcomes of semen volume was contradictory [20, 21]. The results of this study show that ALA could improve the quality of male sperm in clinical practice.

Normally, the oxygen used in our body will produce and release pro-oxidant free radicals, which are neutralized by the intervention of antioxidants inside and outside the cells. Many *in vitro* factors will also lead to the increase of free radicals, break the oxidation-antioxidation balance in the body, and lead to the state of oxidative stress, which will lead to the reduction in the fluidity and deconstruction of the cell membrane (lipid oxidation). Sperm may have structural and genomic changes and DNA fragmentation, which would affect the sperm function and quality, finally damage the fertilization ability [22]. ALA can work equally well in fat-soluble or water-soluble states, intracellular or extracellular media, which is different from other antioxidants that only work in one of the conditions, provide a broader antioxidant activity (broad spectrum) to reduce the adverse effects of ROS. ALA also plays a role by participating in the production of other antioxidants such as glutathione and the regulation of adenosine triphosphate (ATP) [23]. A shield, which is actively produced by ALA over the sperm midpiece through interrelation with glutathione and ascorbic acid, protects the inner organelles from ROS induced by free radicals [24]. In turn, this shield will indirectly reduce the formation of deep pores and cracks on the sperm surface, thus protecting the external and internal structural integrity of the organelle [21]. Sperm motility is highly dependent on mitochondrial ATP activity, while ALA, as a mitochondrial co-enzyme, enhances membrane capacity by augmenting cytochrome C concentrations, thereby increasing availability of ATP, and to ensure constant yield of ATP [25].

Haghighian 2015 [21] did not directly mention the WHO criteria edition in the published article. We obtained the specific WHO semen examination results information from the references, which correspond to different indicators in the study: the semen samples were analyzed according to the WHO criteria 1992 [26], motility assessment of sperm was performed according to WHO criteria 1992 and 1999 [11]. Because the data of the two WHO versions have little difference and according to the specific data involved in the literature, this paper made statistical analysis according to WHO criteria 1999. In addition, Raaia 2012 [13] and Abbasi 2020 [20] adopt WHO 2010 semen examination results [12], because the data types involved in the whole meta-analysis had no methodological differences (including semen volume, sperm concentration, sperm motility, sperm progressive motility, no sperm morphology, WHO 1999 and 2010 had no significant methodological differences, only some statistical differences), so they were included and analyzed together, which would not affect the results of this meta-analysis, The results were still reliable. We listed the methodology of indicators of this meta-analysis in WHO criteria 1992, 1999 and 2010, as shown in the Table 5.

**Table 5:** Methodological differences between the three versions of WHO guidelines

Parameters	Semen Volume	Sperm Concentration	Sperm Motility	Sperm Morphology
WHO guildline of 1992 (Third Edition) [26]	The volume of the ejaculate should be measured either in a graduated cylinder with a conical base or by aspirating the whole sample into a wide mouthed pipette by means of a mechanical device	The concentration of spermatozoa should be determined using the haemocytometer method, containing more than 100 × 10^6^ spermatozoa/ml, a 1:50 dilution may be appropriate. White-blood-cell pipettes and automatic pipettes relying upon air displacement are not accurate enough for making volumetric dilutions of such viscous material as semen. White-blood-cell pipettes and automatic pipettes relying upon air displacement are not accurate enough for making volumetric dilutions of semen. A positive-displacement type of pipette should be used	Each spermatozoa is graded 'a', 'b', 'c', or 'd'. Usually four to six fields have to be scanned to classily 100 successive spermatozoa, yielding percentage for each motility category. The count of 100 spermatozoa repeated and the average values calculated for each category	At least 100, and preferably 200, spermatozoa are counted. With stained preparations, a 100 × oil-immersion bright-field objective without a phase ring should be used
WHO guildline of 1999 (Fourth Edition) [11]	The volume of the ejaculate may be measured using a graduated cylinder with a conical base or by weighing standard containers with and without semen	The concentration of spermatozoa should be determined using the haemocytometer method on two separate preparations of the semen sample, one for each side of the counting chamber. The dilution is determined (1:5, 1:10, 1:20, 1:50) from the preliminary estimation of sperm concentration. White-blood-cell pipettes and automatic pipettes relying upon air displacement are not accurate enough for making volumetric dilutions of semen. A positive-displacement type of pipette should be used scanning the slide and estimating the number of spermatozoa per field or part of a field equivalent to 1 nl gives an approximate sperm concentration in 10^6^/ml. This estimate is used to decide the dilution for determining the sperm concentration by haemocytometry: < 15 spermatozoa, dilution 1:5;15–40 spermatozoa, dilution 1:10;40–200 spermatozoa, dilution 1:20; > 200 spermatozoa, dilution 1: 50	At least five microscopic fields are assessed in a systematic way to classify 200 spermatozoa. The motility of each spermatozoa is graded 'a', 'b', 'c', or 'd', according to whether it shows	With stained preparations, a 100X oil-immersion bright-field objective and at least a 10X ocular should be used. At least 200 consecutive spermatozoa are counted (assessing 200 once is better than l00 twice). Although it is preferable to count 200 spermatozoa twice to reduce counting error and variability
WHO guildline of 2010 (Fifth Edition) [12]	The volume is best measured by weighing the sample in the vessel in which it is collected. Alternatively, the volume can be measured directly. 1.Collect the sample directly into a modified graduated glass measuring cylinder with a wide mouth. These can be obtained commercially 2. Read the volume directly from the graduations	The concentration of spermatozoa in semen is their number (N) divided by the volume in which they were found, i.e. the volume of the total number (n) of rows examined for the replicates. Sperm count > 101 per 400 × field of view, > 404 per 200 × field of view, dilution 1:20; sperm count 16–100 per 400 × field of view; 64–400 per 200 × field of view, dilution 1:5; sperm count 2–15 per 400 × field of view; 8–60 per 200 × field of view, dilution 1:2; sperm count < 2 per 400 × field of view Dilution 1:5; < 8 per 200 × field of view	Examine the slide with phase-contrast optics at × 200 or × 400 magnification. Assess approximately 200 spermatozoa per replicate for the percentage of different motile categories. A simple system for grading motility is recommended that distinguishes spermatozoa with progressive or non-progressive motility from those that are immotile. The motility of each spermatozoa is graded as follows: Progressive motility (PR); Non-progressive motility (NP) and Immotility (IM)	Examine the slide using bright field optics at × 1000 magnification with oil immersion. Evaluate at least 200 spermatozoa in each replicate, in order to achieve an acceptably low sampling error. Repeat the assessment of at least 200 spermatozoa, preferably on the replicate slide, but alternatively on the same slide

*Legend:* We compared the methodological differences between the three versions of WHO guidelines from four aspects, including semen volume, sperm concentration, sperm motility and sperm morphology(that were normal sperm froms and abnormal forms)

*WHO* World Health Orgnization

The ALA group data of progressive mobility in Table 4 post-varicocelectomy/post-medication semen parameters in Abbasi 2020 [20] was 35.75 ± 396. According to the content and data analysis of the previous and subsequent articles, this should be a printing error, and the original data should be 35.75 ± 3.96. Therefore, correct data were used for analysis in our meta-analysis. In this study, semen parameters were measured before operation. After operation, participants were randomly divided into ALA group and placebo group. Semen parameters were checked after 80 days of treatment. The results of this study give us a very interesting results. There was no significant statistical difference between ALA group and control group except one group of data (abnormal sperm forms). In other words, the existence of varicocele, a basic disease, did not seem to affect the research results of the trial. In general, there was little difference between ALA and placebo group. Although it did not affect this systematic review and meta-analysis, it was recommended to reduce the impact of basic diseases on drug experiments in subsequent experiments, so as to draw more reliable conclusions.

The sensitivity analysis (see in Table 3) showed that, Raaia 2012 [13] (3rd month) and Haghighian 2015 [21] were found to affect the overall prevalence estimate. Because in these two studies, the change of sperm concentration between the treatment group and the control group was the largest, with significant statistical difference. For the rest three groups of data, there were no significant difference in sperm concentration. The sensitivity analysis (see in Table 4) showed that, Abbasi 2020 [20] was found to affect the overall prevalence estimate. Because the patients in this study had different degrees of varicocele, ALA and placebo treatment were performed after operation of varicocelectomy. The difference between the treatment group and the control group was very obvious, which affects the overall level of the whole group data.

The study Raaia 2012 [13] included in this meta-analysis did not mention the detailed random grouping method in the original text. In order to ensure the rigor of the study, we sent an e-mail to the author to ask about relevant questions, but we didn't get a reply from the authors.

Through the previous literature search, we found another very valuable study Rago 2017 [22]. The results of semen parameters in this article were described as follows: the statistical analysis of the pre- and post-treatment seminal parameters did not reveal any statistically significant differences. Because there was no particular data, we sent an e-mail to the corresponding author asking for the original data for this system review, but unfortunately we didn't get a reply from the author.

In addition, another study, Hodeeb 2022 [23], was retrieved this time. Because this was an observational study and did not belong to RCT, it could not be included in this meta-analysis. Among them, semen volume(*p* < 0.001), sperm concentration(*p* < 0.0001), total mobility(*p* < 0.001), progressive mobility(*p* < 0.001) and sperm vitality(*p* < 0.001) were significantly improved after treatment, but sperm morphology(*p* = 0.064) had no significant difference.

At present, the most commonly used alpha-lipoic acid in China is WEI YI NENG® (Alpha-lipoic acid capsules, also called Thioctic Acid Capsules, JS.WANHE PHARM, Jiangsu Province, China).WEI YI NENG® is used to treat diabetes multiple peripheral neuropathy. In recent years, WEI YI NENG® is also widely used in the treatment of male infertility, the dosage is 600 mg per day (200 mg three times a day, or 300 mg two times a day). It has a good effect in improving normal sperm forms [27] and reducing sperm DNA fragments [28].

The existing systematic reviews searched and included studies before May 2020 [8], with the types of observational studies (such as cohort or case control studies), RCTs, prospective clinical trials and case reports, so the level of evidence-based medicine was slightly lower. Our study differs in that it is the first time to include only RTCs on the treatment of sperm with ALA, demonstrating a significant clinical and statistical improvement in some sperm paremeters, and thus can be regarded as level 1A evidence, which has important clinical value.

Although our study has important strengths, there were still limitations exist. Only three studies included; all included trials had small samples; the largest study included in our meta-analysis had only 48 men; one of the studies [13] did not inform us the registration information of the clinical trial; follow-up was limited to approximately 3 months in all studies and every month's data had been provided by only one study [13].

## Conclusions

In this meta-analysis of RCTs, normal sperm forms, sperm concentration, sperm total motility and progressive motility have improved in men treated with ALA compared to men treated with sham treatment. However, before this treatment option is widely accepted, more stringent and larger sample RCTs must be conducted.

## Data Availability

All the data and material are available in the manuscript.

## Abbreviations

ALA: Alpha-lipoic Acid
ATP: Adenosine Triphosphate
CBM: China Biology Medicine Database
CI: Confidence interval
CNKI: China National Knowledge Infrastructure Database
MD: Mean difference
PICO: Population, interventions, comparisons, outcomes
PRISMA: Preferred Reporting Items for Systematic Reviews and Meta-Analyses
RCT: Randomized Controlled Trial
ROS: Reactive Oxygen Species
SD: Standard deviation
SE: Standard error
WHO: World Health Organization
